# Pericardial agenesis: a case report of a rare congenital heart disease

**DOI:** 10.1093/ehjcr/ytae200

**Published:** 2024-04-18

**Authors:** Giancarlo Trimarchi, Concetta Zito, Giuseppe Pelaggi, Scipione Carerj, Gianluca Di Bella

**Affiliations:** Department of Clinical and Experimental Medicine, Cardiology Unit, University of Messina, AOU G.Martino, Via Consolare Valeria, 98125 Messina, Italy; Department of Clinical and Experimental Medicine, Cardiology Unit, University of Messina, AOU G.Martino, Via Consolare Valeria, 98125 Messina, Italy; Department of Clinical and Experimental Medicine, Cardiology Unit, University of Messina, AOU G.Martino, Via Consolare Valeria, 98125 Messina, Italy; Department of Clinical and Experimental Medicine, Cardiology Unit, University of Messina, AOU G.Martino, Via Consolare Valeria, 98125 Messina, Italy; Department of Clinical and Experimental Medicine, Cardiology Unit, University of Messina, AOU G.Martino, Via Consolare Valeria, 98125 Messina, Italy

**Keywords:** Pericardial agenesis, Pericardial disease, Congenital heart disease, Cardiac magnetic resonance, Case report

## Abstract

**Background:**

Pericardial agenesis is a rare congenital heart disease characterized by a variable clinical presentation.

**Case summary:**

A 32-year-old man was sent by an occupational health physician to our health care centre because of pathological electrocardiogram (ECG). On transthoracic echocardiogram, we had some difficulty to obtain a good quality of four-chamber apical view that was shifted upper and laterally towards the left anterior axillary line. Nonetheless, an abnormal diastolic expansion of the apex of the left ventricle (LV) that had an otherwise normal systolic function was detected. A chest X-ray confirmed the leftward shift of the heart, with the elongation of the left border of cardiac silhouette and cardiac MRI, finally revealed the absence of left-sided pericardium associated with a leftward dislocation of the heart and a dysmorphism of the LV apex that appeared rounded and curved. The non-invasive work-up was completed with 48 h long Holter ECG that was unremarkable. The exercise test was also negative for both inducible myocardial ischaemia and arrhythmias. Patient was scheduled for loop-recorder implantation, and a 6-month clinical follow-up was advised.

**Discussion:**

Pericardial agenesis is a rare congenital heart disease associated with an increased risk of cardiac arrhythmias and type A aortic dissection, however its clinical course could be also completely unremarkable. The diagnosis is challenging, and cardiac MRI remains the gold standard imaging modality. In complete left-sided and asymptomatic forms, no treatment is needed. Prognosis is not well established due to both the rarity of disease and extreme variability of clinical presentation.

**Learning points**

- Due to the lack of deep knowledge and unremarkable clinical course, congenital pericardial agenesis is commonly misdiagnosed.
- The diagnosis is greatly aided by the direct and indirect signs of pericardial abnormalities that are shown by cardiac imaging modalities such as echocardiography, X-ray imaging, and cardiac magnetic resonance.
- Patients with congenital pericardial agenesis are often asymptomatic and, generally, do not need treatment. Surgery is mainly used to treat patients with partial forms who might develop complications like herniation of heart chambers, type A aortic dissection, or coronary artery compression.

## Introduction

Congenital absence of the pericardium is an extremely rare form of pericardial defect, with an approximate incidence of <1:10 000, due to the failure of pleuro-pericardial membranes to fuse completely on one or both sides.^1,2^ Pericardial agenesis can be classified into two forms: complete and partial. Complete form is the most frequent and it can be bilateral, left-sided, and right-sided. On the other hand, partial form is extremely rare and is associated with an increased risk of complications.^3^ About 30–50% of patients with a congenital absence of the pericardium may have other congenital heart disease such as atrial septal defects, patent ductus arteriosus, and tetralogy of Fallot. It has also been described in patients with Marfan syndrome and aortic connective tissue disorders. Additionally, non-cardiac anomalies such as pectus excavatum, diaphragmatic hernia, and associations with VACTERL (Vertebrae-Anus-Cardiac-Trachea-Esophagus-Renal, Limbs&radius) syndrome and Pallister–Killian syndrome were reported.^4^

## Summary figure

**Table:** 

Timepoint	Clinical description
Presentation	Health surveillance visit from the occupational physician with evidence of electrocardiographic abnormalities.
Day 7	Visit at our Cardiology Clinic and transthoracic echocardiogram with evidence of altered shape of the left ventricle characterized by a bulge of the apical segments.
Day 9	X-ray was performed with evidence of leftward shift of the heart and elongation of the left border of cardiac silhouette.
Day 30	Cardiac MRI was performed with final diagnosis of complete left-sided pericardial agenesis.
Day 35	Holter ECG registration for a length of 48 h with unremarkable results for major ventricular events and coronary cardiac computed tomography that was negative.
Day 40	The patient was scheduled for loop-recorder implantation and a 6-month clinical follow-up.

## Case presentation

**Figure 1:**
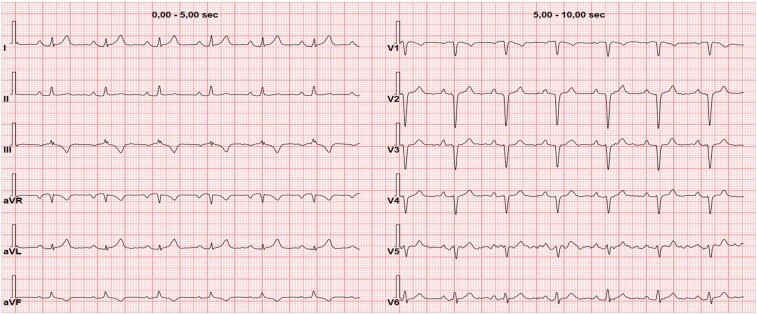
ECG showing sinus rhythm with 70 b.p.m., low voltages in peripheral leads, normal atrioventricular conduction, absence of R-wave progression in the precordial leads, and prominent negative T waves in III and AVF leads.

An asymptomatic 32-year-old Caucasian man with no medical history and cardiac risk factors (except for class 2 obesity, body mass index 36 kg/m^2^) was sent by an occupational health physician to our health care centre because of electrocardiographic (ECG) alterations consisting in low voltages of peripheral leads, absence of R-wave progression in the precordial leads, and prominent negative T waves in III and AVF leads (*Figure 1*). Familiar anamnesis was negative and free from cardiac events. Physical examination did not show any significant alterations, except for a systolic ejection murmur at the left sternal border. No oedema or congestion signs were found.

**Figure 2:**
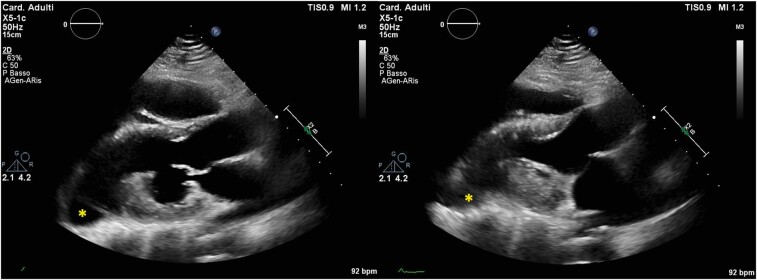
Transthoracic echocardiogram, parasternal long axis view (LAX): on the left, end-diastolic frame showing an unusual shape of the left ventricle (LV) with a sharp interruption of mid segments visualization and a bulging of the apical segments (asterisk), normally undetectable on parasternal LAX view; on the right, end-systolic frame showing a normal wall thickening of the basal and mid segments of the LV with a systolic expansion of the virtual apex (yellow asterisk).

**Figure 3:**
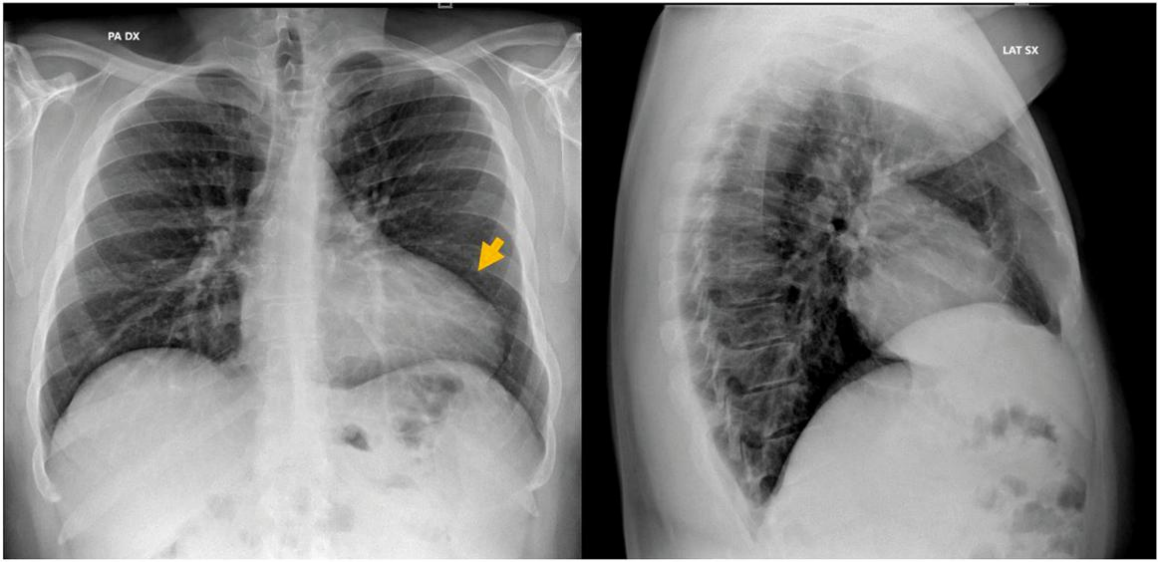
Chest X-ray PA and LL view, showing the heart rotating backwards and to the left as the left edge of the heart silhouette is straightened and elongated (Snoopy sign, arrow).

**Figure 4:**
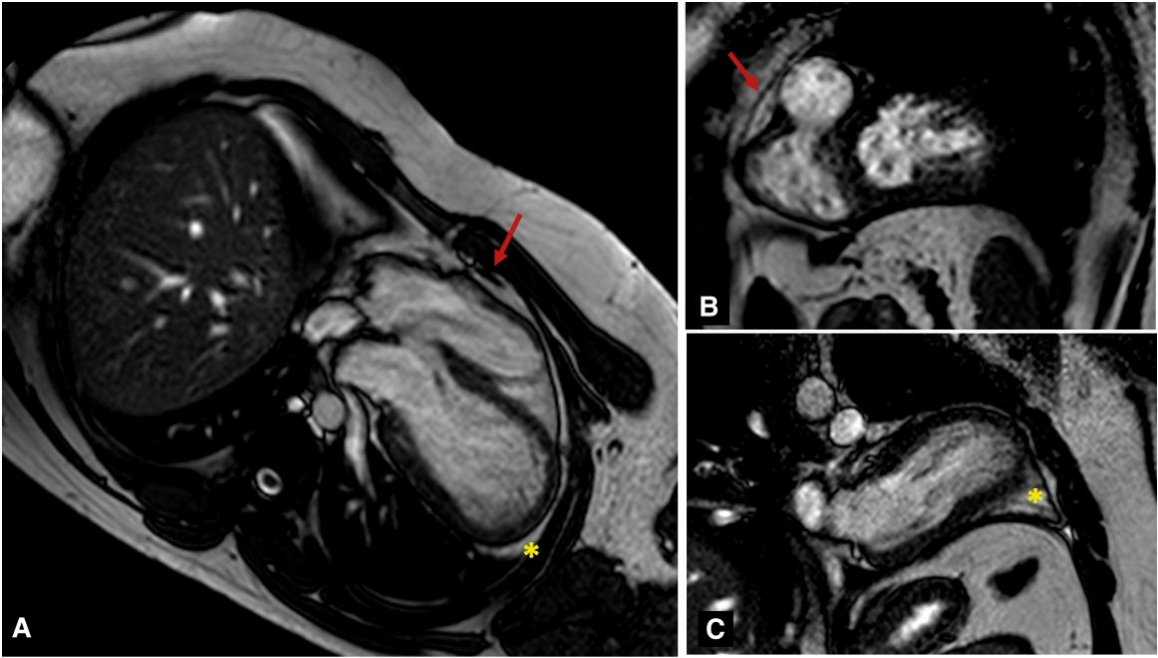
Cardiac MRI: (*A*) four-chamber view showing dislocation of the heart towards the left hemithorax and presence of right pericardium at basal level (arrow); (*B*) short axis view showing the presence of pericardium along the anterior wall of the right ventricle (arrow); (*C*) two-chamber view showing complete pericardium absence along the left ventricle (asterisk).

Transthoracic echocardiographic examination was immediately performed showing an altered shape of the left ventricle (LV) characterized by a bulge of the apical segments, on the parasternal long axis view, obtained in both standard 2D (*Figure 2*) and X-plane modality. There was also a difficult visualization of the heart from a standard four-chamber apical view (see Supplementary material online, Video S1) that was shifted upper and laterally towards the left anterior axillary line. Furthermore, LV function analysis revealed an abnormal diastolic expansion of the apex with normal systolic wall motion and definitively preserved LV ejection fraction (EF 55%). To confirm the unusual position of the heart in the mediastinum and to study its spatial relationship with adjacent structures, we submitted patient to chest X-ray (*Figure 3*). This proved the leftward shift of the heart, with the elongation of the left border of cardiac silhouette (‘Snoopy sign’)^5^ and further showed the interposition of lung tissue (seen as a black band) between the base of the heart and the left hemidiaphragm.^4^ Therefore, owing to the presence of radiological findings suggestive for pericardial agenesis, a cardiac magnetic resonance imaging (MRI) was performed, confirming the diagnosis of complete left-sided pericardial agenesis. Particularly, MRI revealed the absence of pericardium, the leftward dislocation of the heart, and the dysmorphism of the LV apex that appeared rounded and curved (*Figure 4*) and an excessive movement of the cardiac apex (see Supplementary material online, Videos S2 and S3), all typical signs of this cardiac congenital abnormality. Moreover, ventricles had normal systolic function and were non-dilated as demonstrated by normal indexed volumes, mass and EF {LVEDVi = 67 mL/m^2^ [normal reference range (nrr) 57–105]; LVESVi 30 mL/m^2^ [nrr 14–38]; LVMi = 64 g/m^2^ [nrr 49–85]; SVi = 37 mL/m^2^ [nrr 33–72]; LVEF = 57% [nrr 56–77]; right ventricle: RVEDVi = 68 mL/m^2^ [nrr 61–121]; RVESVi 24 mL/m^2^ [nrr 19–59]; SVi = 33 mL/m^2^ [nrr 28–75]; RVEF = 57% [nrr 52–72]}. Otherwise, there were no signs of inflammatory heart diseases, as demonstrated through T2-weighted sequences not showing myocardial oedema and through the absence of late gadolinium enhancement at the level of the myocardium of both ventricles. Coronary anatomy was not documented with cardiac MRI because of its technical limitations, including reduced spatial resolution, longer acquisition times, and lower signal-to-noise ratios in comparison with cardiac computed tomography. The laboratory tests were within normal ranges.

Owing to the knowledge that pericardial agenesis could be associated with cardiac arrhythmias, we completed our non-invasive work-up with a Holter ECG registration for a length of 48 h and the results were unremarkable for major ventricular events. Furthermore, patient underwent exercise test that did not reveal any inducible myocardial ischaemia and dangerous arrhythmias. However, patient underwent non-invasive coronary angiography by cardiac computed tomography revealing normal coronary tree. Finally, after multidisciplinary team evaluation, patient was scheduled for a loop-recorder implantation, and a 6-month clinical follow-up was advised.

## Discussion

Pericardial agenesis is a rare congenital heart disease characterized by a non-specific clinical course and it is often asymptomatic, as presented in our case. The absence of the pericardium can cause cardiac chambers to dislocate, contacting nearby structures, potentially triggering extrasystoles or severe arrhythmias. Additionally, heart hypermobility has been linked to a heightened risk of type A aortic dissection, underlining the critical role of the pericardium in stabilizing heart position and function.^6,7^

The diagnosis is challenging and is generally suspected during cardiological screening performed for other reasons. A chest X-ray can demonstrate a marked leftward deviation of the heart. ECG anomalies (absence of R-wave progression, QRS axis deviation, right branch block in right side forms) are not specific. Despite the lack of specificity, the following echocardiographic abnormalities may at least raise the suspicion of pericardial agenesis: atypical acoustic windows, which may indicate displacement of the heart, right ventricular dilatation, excessive cardiac apex motion, and paradoxical septal motion during systole.^3^ However, cardiac MRI remains the gold standard imaging modality for the diagnosis.^8,9^ Indeed, it is more sensitive than echocardiography in detecting indirect indicators of pericardial defects, such as excessive heart laevorotation, pulmonary tissue interposition between the aorta and the main pulmonary artery, or between the heart’s underside and the diaphragm.^10^ Particularly, cardiac MRI can make use of cine sequences to visualize functional heart abnormalities more clearly, such as the excessive movement of the cardiac apex, which is often linked to the lack of the pericardium.^11^

With regard to the therapy, it has been already demonstrated that in complete and asymptomatic forms, as in this case, no treatment is needed, and the therapeutic choice is a long-term follow-up in order to estimate the progression of the disease.^12^ Otherwise, patients with partial absence of pericardium are at higher risk of herniation and strangulation of cardiac and vascular structures.^12^ The risk of complications is related to the site of partial pericardial defect and there is still no agreement on which form requires preventive intervention.^4^ Basically, partial form requires more attention and a closer follow-up than complete pericardial agenesis. Surgery is advised in the presence of serious complications or in case of symptoms, more commonly persistent and intense chest pain. Some surgical options could be the closure of pericardial foramen, pericardiectomy or pericardioplasty.^13^ Prognosis is still not well established due to both the rarity of the disease and extreme variability of clinical presentation.

In conclusion, we presented the case of an asymptomatic 32-year-old obese man affected by complete left-sided pericardial agenesis with a good prognosis, who did not need surgical treatment. In this case, a comprehensive diagnostic work-up with multimodality imaging played a key role to avoid diagnostic errors and to optimize patient follow-up to prevent complications in the future.

## Supplementary Material

ytae200_Supplementary_Data

## Data Availability

The data underlying this article will be shared on reasonable request to the corresponding author.

## Lead author biography

Giancarlo Trimarchi was born in Messina, Italy, in 1995. He graduated as a medical doctor in School of Medicine of Catania University in 2020. He is currently a cardiology resident in the Department of Clinical and Experimental Medicine of University of Messina. He has a special interest for myocardial and pericardial diseases.

## Supplementary material

**Consent:** The authors confirm that written consent for submission and publication of this case report including image(s) and associated text has been obtained from the patient in line with COPE guidance.

**Funding:** None declared.
